# An Institutional Shift from Routine to Selective Diversion of Low Anastomosis in Robotic TME Surgery for Rectal Cancer Patients Using the KHANS Technique: A Single-Centre Cohort Study

**DOI:** 10.3390/jpm14070725

**Published:** 2024-07-04

**Authors:** Rauand Duhoky, Guglielmo Niccolò Piozzi, Marieke L. W. Rutgers, Ioannis Mykoniatis, Najaf Siddiqi, Syed Naqvi, Jim S. Khan

**Affiliations:** 1 Department of Colorectal Surgery, Portsmouth Hospitals University NHS Trust, Queen Alexandra Hospital, Southwick Hill Road, Cosham, Portsmouth PO6 3LY, UK; rauand.duhoky@porthosp.nhs.uk (R.D.); guglielmo.piozzi@porthosp.nhs.uk (G.N.P.);; 2 School of Computing, Faculty of Technology, University of Portsmouth, Portsmouth PO1 2UP, UK; 3 Faculty of Science and Health, University of Portsmouth, Portsmouth PO1 2UP, UK

**Keywords:** rectal cancer, robotic surgery, stoma, selective diversion, TME, anastomosis

## Abstract

(1) Background: In recent years, there has been a change in practice for diverting stomas in rectal cancer surgery, shifting from routine diverting stomas to a more selective approach. Studies suggest that the benefits of temporary ileostomies do not live up to their risks, such as high-output stomas, stoma dysfunction, and reoperation. (2) Methods: All rectal cancer patients treated with a robotic resection in a single tertiary colorectal centre in the UK from 2013 to 2021 were analysed. In 2015, our unit made a shift to a more selective approach to temporary diverting ileostomies. The cohort was divided into a routine diversion group treated before 2015 and a selective diversion group treated after 2015. Both groups were analysed and compared for short-term outcomes and morbidities. (3) Results: In group A, 63/70 patients (90%) had a diverting stoma compared to 98/135 patients (72.6%) in group B (*p* = 0.004). There were no significant differences between the groups in anastomotic leakages (11.8% vs. 17.8%, *p* = 0.312) or other complications (*p* = 0.117). There were also no significant differences in readmission (3.8% vs. 2.6%, *p* = 0.312) or reoperation (3.8% vs. 2.6%, *p* = 1.000) after stoma closure. After 1 year, 71.6% and 71.9% (*p* = 1.000) of patients were stoma-free. One major reason for the delay in stoma reversal was the COVID-19 pandemic, which only occurred in group B (0% vs. 22%, *p* = 0.054). (4) Conclusions: A more selective approach to diverting stomas for robotic rectal cancer patients does not lead to more complications or leaks and can be considered in the treatment of rectal cancer tumours.

## 1. Introduction

Current practice in restorative rectal cancer surgery involves the creation of a (temporary) protecting diverting stoma with the purpose of minimising the severe septic consequences of an anastomotic leakage (AL). The presentation of ALs can vary, with some being clinically symptomatic despite the presence of a proximal stoma. Symptomatic ALs will require intervention, whilst asymptomatic leaks are only diagnosed radiologically later, prior to reversal, and do not necessarily require intervention.

The incidence of AL is around 20% and is associated with severe complications and morbidity, reoperations, and mortality [1,2,3,4]. AL has a significant impact on oncological outcomes and quality of life and is associated with significant healthcare costs [5,6,7,8,9,10,11].

Furthermore, the stoma itself can lead to significant discomfort, low self-image, decreased quality of life, dehydration, electrolyte imbalance, and other complications leading to readmission and/or reoperation [12,13]. The presence of a stoma also necessitates costs for appliances, guidance from stoma nurses, and another surgical intervention (the stoma reversal), which increases the risk of an incisional hernia [14]. After a 3-month period in which the anastomosis is deemed to have healed sufficiently, the stoma is usually reversed. This 3-month period is often exceeded due to delaying factors such as complications, adjuvant therapy, pressures on surgical waiting lists, a second surgical procedure, and late discoveries of (asymptomatic) AL.

In recent years, some centres have shifted from routine diversion (RD) towards a more selective use of diversion (SD), as more evidence seems to suggest that the benefits of RD may not outweigh its risks and could lead to an even higher permanent stoma rate with comparable AL rates [9,15,16]. Recent studies have suggested that the presence of an ileostomy does not reduce the incidence of AL but only delays the diagnosis, with possibly reduced possibilities for restoring the anastomosis [9,17,18].

The advantages of a robotic platform for low rectal resection include the use of an advanced robotically controlled stapler for rectal transection that is able to evaluate and adapt stapling according to tissue thickness (i.e., Sureform, Intuitive Surgical, Sunnyvale, CA, USA), easy assessment of perfusion using indocyanine green (ICG) dye with the Firefly mode, and the possibility to easily perform suture reinforcements of the transverse rectal staple line at the superior and inferior ends (i.e., ‘dog ears’) and of the circular stapler line using interrupted sutures. This was described as the ‘KHANS’ technique (Key enhancement of the Anastomosis for No-Stoma Surgery) and was developed in 2015, aiming to reduce the incidence of AL and the need for diverting stomas in robot-assisted rectal resections [19].

This study aims to evaluate the impact of shifting from RD to SD in a tertiary oncological colorectal centre by assessing the prevalence of AL and stoma-related outcomes between these groups.

## 2. Materials and Methods

### 2.1. Study Design

This study retrospectively evaluated a consecutive series of rectal cancer resections with primary anastomosis performed between 2013 and 2021. Data were extracted from a prospectively maintained colorectal database containing data from three robotic and three laparoscopic colorectal surgeons. All surgeons were past their learning curves for their respective approaches at the time of data capture (estimated at 35 procedures based on the existing literature) [20,21]. The switch from RD to SD was made in 2015. Two groups were defined and compared: the RD group (2013–2015) and the SD group (2016–2021).

The primary endpoint was to report on complication rate, including AL (with ISREC classification) [10]. Secondary endpoints included reporting length of stay (LOS), stoma rate after 1 year, readmissions < 31 days, and reoperations < 31 days.

Inclusion criteria were the following: (1) robotic approach; (2) rectosigmoid cancer resection; and (3) availability of stoma follow-up data.

Exclusion criteria were the following: (1) synchronous cancers; (2) abdominoperineal resection; (3) open approach; (4) hand-sewn anastomosis; (5) permanent colostomy; (6) lost to follow-up within a year.

Medical ethical approval was acquired from the Health Research Authority in the United Kingdom, and it was judged that written informed consent would not be necessary for this study because of its retrospective nature and subsequent anonymous analysis of the data.

### 2.2. Procedure

Preoperative diagnostics included patient demographics, tumour histology and staging, computed tomography (CT) scans of the chest, abdomen, and pelvis, and magnetic resonance imaging (MRI) of the pelvis. Indications for neoadjuvant chemoradiation (neoCRT) were tumours with threatened/suspicious circumferential resection margin (CRM). Patients underwent short-course or long-course neoCRT after multidisciplinary team discussion, with clinical restaging and surgery performed 10–12 weeks thereafter. Neoadjuvant protocols in the neoCRT group were either short-course neoadjuvant radiotherapy (25 Gy in 5 fractions over 5 weekdays) or long-course neoadjuvant chemoradiotherapy (45–50 Gy in 25 fractions over 5 weeks), with concomitant chemotherapy (3 months of CAPOX (capecitabine and oxaliplatin) or FOLFOX (folinic acid, fluorouracil, and oxaliplatin)). Adjuvant therapy consisted of 45 Gy in 25 fractions. All patients underwent preoperative mechanical bowel preparation.

All operations were performed on the da Vinci Si/X/Xi^®^ platform according to availability. A five-port single-docking fully robotic approach with a two-left-hand setting was used [22]. Before transection of the rectum, an intravenous bolus of ICG (3 mL, 7.5 mg) was administered to identify the perfusion status of the rectum. Optimal level of transection was decided upon consensus between surgeon and assistant. Where patients did not receive a stoma, the KHANS (Key enHancement of the Anastomosis for No Stoma) technique was used to reinforce the anastomosis [19].

In the RD group, patients predominantly received a diverting stoma, and the few instances where they were treated with a primary anastomosis were decided based on surgeon preference and opinion. In the SD group, the following factors were used to help determine whether a patient would not receive a diverting stoma: (1) medical and nutritional status (such as ASA < 4); (2) not requiring multivisceral resection or colo-anal anastomosis; and (3) non-obstructive cancer. In cases of diverting stoma formation, the anastomosis was checked twice prior to reversal: with gastrografin enema imaging and with sigmoidoscopy under anaesthesia at the time of reversal.

### 2.3. Outcomes

Clinical and oncological outcomes were recorded, including AL, which was categorised according to the ISREC classification [10] into type A, requiring no active intervention; type B, requiring an active therapeutic intervention, such as antibiotics or radiologic drainage; and type C, necessitating a return to theatre. ALs were classified as early leaks when discovered <30 days after surgery and late leaks when discovered >30 days post-surgery. Postoperatively, all patients were managed with the same enhanced recovery programme (ERAS) according to NHS standards, with a liquid diet for the first 48 h and regular laxatives (lactulose 10 mL twice a day) starting from the 3rd postoperative day [23].

### 2.4. Statistical Analysis

Baseline characteristics were analysed for each group, including means and standard deviations for normally distributed data and medians and interquartile ranges for non-normally distributed data. Bivariate categorical data were analysed using Chi-square or Fisher’s exact test. Numerical data were analysed using either unpaired *t*-test or Mann–Whitney U test, depending on the distribution of data. Time-to-event (time to anastomotic failure and stoma-free survival) data were compared between the groups using the log-rank test (with Kaplan–Meier curves) or cox regression analysis. In the case of a secondary stoma, time was calculated from the date of surgery to the reversal of the second stoma. A *p*-value < 0.05 was considered statistically significant. All analyses were performed using IBM SPSS Statistics for Windows, version 28.0.0 (IBM Corp., Armonk, NY, USA). The STROBE guidelines for observational studies were followed [24].

## 3. Results

### 3.1. Patient Characteristics

**Figure 1:**
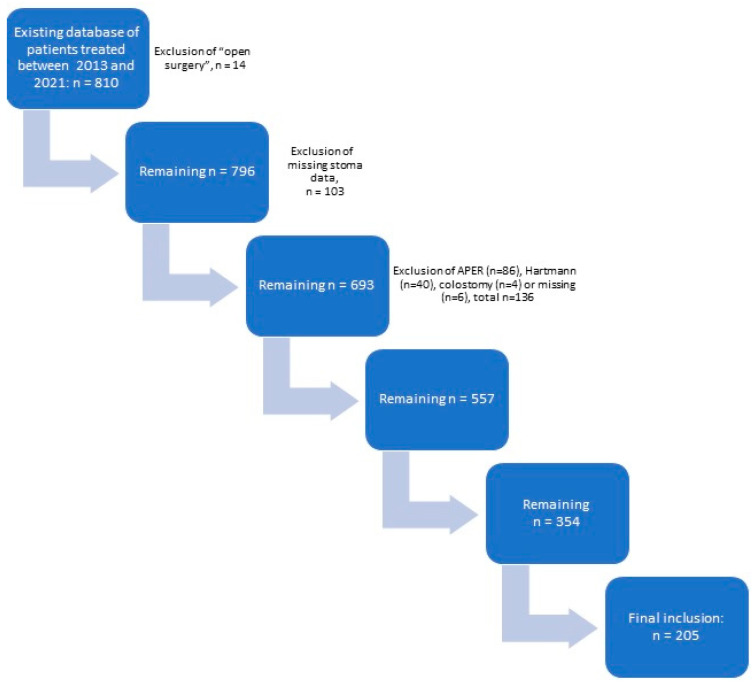
Flow diagram of patient selection. APER = Abdominoperineal Excision of the Rectum.

A total of 205 robotic rectal cancer patients were enrolled in this study (see Figure 1): group A (RD), *n* = 70; and group B (SD), n = 135. The baseline characteristics are described in Table 1. Both groups had mostly male patients, with a median age of 68.00 [62.75–74.25] vs. 69.00 [58.00–76.00] years. Most patients were ASA II. Group A had significantly more neoadjuvant chemoradiotherapy (17.1% vs. 7.4%, *p* = 0.011). Group B had more advanced tumours (T3/T4: 39.1% vs. 57.8%). The median tumour height measured on preoperative MRI was between 5 and 6 cm from the anorectal junction. There were no significant differences in other baseline variables.

### 3.2. Operative Outcomes

Table 2 shows the operative and postoperative details. There were no conversions in both robotic cohorts. There was a statistically significant difference in stapler use, with group A using the laparoscopic stapler significantly more (95.7% vs. 32.0%, *p* < 0.001). Group A did not employ the robotic stapler at all, and it was used in 67.2% of cases for group B. There was also a significant difference in the number of firings, with group B having significantly fewer linear firings than group A (*p* < 0.001). The operative time was 15 min shorter in group A, with 240 min vs. 255 min in group B (*p* = 0.029). The operative time in group A was significantly shorter (240 vs. 255 min, *p* = 0.029). No cases involved more than three staple firings in group B. The LOS was comparable between the cohorts, with a median of 6 days (*p* = 0.292). The complication rates (*p* = 0.117), readmission rates (15.9% vs. 17.0%, *p* = 1.000), pathological T-staging (*p* = 0.623), pathological R-staging (4.3% vs. 5.2%, *p* = 1.000), reoperation rates (2.9% vs. 6.7%, *p* = 0.342), and 90-day mortality rates (*p* = 1.000) were all comparable between groups.

### 3.3. Anastomotic Leakage

The complication rates and AL rates were comparable between the two cohorts (see Table 2). The AL rates were comparable, with 11.8% in group A vs. 17.8% in group B, *p* = 0.312. AL details are described in Table 3. Of the class C leaks, the majority were managed with reoperation, and one in group B (14.3%) was managed with radiological drainage. All other leaks were treated conservatively with antibiotics. The median time until leakage was not statistically significantly different between the groups (31.50 [3.75–96.25] days in group A vs. 8.50 [4.00–65.50] days in group B). There was no mortality due to AL in either group.

### 3.4. Stoma Formation

Table 4 displays the stoma-related variables and shows that the later selective diversion cohort, group B, underwent significantly less stoma formation than group A (72.6% vs. 90.0%, *p* = 0.004). There were no statistically significant differences in stoma closure rates (82.5% vs. 78.6%, *p* = 0.686), complications after stoma closure (40.0% vs. 50.8%, *p* = 0.329), LOS after stoma closure (4.00 [3.00–7.00] vs. 4.00 [4.00–6.25], *p* = 0.373), readmission after stoma closure (3.8% vs. 2.6%, *p* = 0.312), reoperation after stoma closure (3.8% vs. 2.6%, *p* = 1.000), secondary stoma rates (3.0% vs. 5.3%, *p* = 0.721), stoma-free survival at 1 year (71.9% vs. 71.6%, *p* = 1.000), or stoma-free survival at the end of follow-up (85.1% vs. 75.9%, *p* = 0.146) between the cohorts. The median time until closure was 284.0 [183.25–378.75] vs. 234.0 [148.00–332.00] days (*p* = 0.307). The most common reasons for delayed reversal were adjuvant chemotherapy (58.8% vs. 40.7%), anastomotic leakage (17.6% vs. 22.2%), and the COVID-19 pandemic.

A subanalysis limiting cases to patients treated before 2019, the pre-COVID era, showed no statistically significant difference in stoma closure between the groups, with 74.3% closure in group A vs. 61.3% in group B (*p* = 0.81) (not displayed).

Table 5 compares the diverted with the non-diverted patients within the selective diversion group B. Less radiotherapy was administered in the no-stoma group compared to the stoma group (5.4% vs. 8.4%, *p* = 0.046), and more adjuvant chemotherapy was administered in the no-stoma group (32.4% vs. 18.4%, *p* = 0.011). The tumours in the no-stoma group were significantly higher at 8 cm from the anorectal junction compared to 5 cm in the stoma group (*p* < 0.001).

The no-stoma group had lower preoperative T-staging compared to the stoma group (*p* = 0.021), with less positive or threatened CRM on MRI (*p* = 0.022), but there were no differences in pathological T- (*p* = 0.164) or R-staging (5.4% vs. 5.1%, *p* = 1.000). The LOS was significantly shorter in the no-stoma group, with 4 days compared to 6 days in the stoma group (*p* < 0.001). The readmission (*p* = 0.309), reoperation (*p* = 1.000), and complication (0.154) rates were comparable. The AL rate was comparable (*p* = 0.081), with 8.1% (n = 3) in the no-stoma group, all of which were early leaks, compared to 21.4% (n = 21) in the stoma group, of which 71.4% were early and 28.6% were late leaks. Of the three leaks in the no-stoma group, one was an ISREC type B leak, and two were type C leaks. In the stoma group, 5 were classified as type A leaks, 11 as type B leaks, and 5 as type C leaks. No mortality was seen in either group (*p* = 1.000).

## 4. Discussion

This observational, retrospective, single-centre cohort study evaluated the institutional change from routine diversion to selective diversion in robotic TME surgery for mid- to low-rectal cancer. We found that the use of selective diversion did not lead to an increase in anastomotic leakage or complications.

Despite a more selective approach to diversion, the selective diversion group did not show a significantly higher rate of stoma-free survival at the end of follow-up. In the present study, the main reasons for delayed reversal were adjuvant treatment, complications, and the COVID-19 pandemic. Because of COVID-19 admissions and reduced theatre time, many patients treated in and after 2019 had delayed reversal of their stomas. This is confirmed in our subanalysis limiting cases to patients treated before 2019, in which the stoma closure rate between the two groups was comparable (*p* = 0.081).

A reoperation and/or intervention is often necessary when an AL occurs. In our study, the AL rate (including both early and late leaks and asymptomatic leaks on gastrografin imaging) varies between 11.8% and 17.8%, with the latter being in group B but not reaching statistical significance; hence, no hard conclusions can be drawn about this outcome. When a stoma is created, a gastrografin imaging study to evaluate the anastomosis is usually performed just prior to reversal. This may lead, due to the reasons mentioned above, to underreporting in the literature when only counting early leaks. More recent studies report both early and late leaks, and leakage rates of 20% appear to be realistic [16,17,25]. Also, anastomotic leakage occurrence seems to be multi-factorial, with one of those factors being a distal staple line [4,26,27,28,29,30,31]. In our study, the median tumour height lies between 5 and 7 centimetres from the anorectal junction, which would be considered a low- to mid-rectum tumour and can be a risk factor for AL. Lastly, a relatively large percentage of the patients included received adjuvant chemotherapy, which is known to influence the wound healing process and be a risk factor for leakages as well.

Another point of interest is the relatively low number of significant (ISREC class C) leaks in the entire study population. The robotic cohort had a total of eight leaks in 205 patients (3.9%), which is low compared to the internationally published literature for mid- to low-rectal cancers. The GRECCAR group reported clinical leaks in 55 out of 449 patients, of which 35 required a reoperation or radiological intervention (7.8%), which would correlate with a class C leak in the ISREC classification [11]. It does need to be mentioned that the patient characteristics described in the GRECCAR group show a higher rate of T3, N1/2, and M1 tumours compared to the present group, as well as a higher preoperative chemoradiotherapy rate (69.5%). The difference in significant leak rates may also be due to the selective use of neoadjuvant chemoradiotherapy in our unit compared to other sites and the use of robotic surgery.

Furthermore, there was no difference in the overall complication rate between the groups, despite a more selective approach to stoma diversion. The fact that there were fewer stomas and less stapling in group B but no difference in complications supports the safety of a more selective approach.

We performed a subanalysis within group B to compare the diverting stomas to the non-diverting stomas after the implementation of a more selective approach. The differences in baseline characteristics found here are consistent with the criteria for SD as described in the Methods section. Although there were significant differences in preoperative T- and CRM-staging, it should be mentioned that there were no significant differences in pathological T- and R-staging, which reduces the risk of bias for these variables. The non-stoma group in this subanalysis had a significantly shorter operative time and LOS than the stoma group. The no-stoma group did not experience significantly more readmissions, reoperations, complications, or mortality than the stoma group, which supports the safety and feasibility of averting a diverting stoma.

When establishing an anastomosis, technique and instruments are key. Using a circular stapler for the anastomosis will cause the lateral areas of the transverse staple line to stand out, commonly referred to as “dog ears”, which can be a potential site for postoperative anastomotic leaks due to tissue ischemia. Colorectal surgeons rely on linear and circular staplers for their anastomoses, and whilst most minimal access surgeons will accept two firings of a linear stapler, stapling in the lower pelvis can be challenging and can require up to four firings to achieve complete rectal division, which might also increase the risk of anastomotic leakage [29,32,33]. It is hypothesised that robotic staplers can reduce the number of firings needed to transect the rectum [34].

In the present study, most robotic stapler rectal transections (n = 63) were successfully completed with two firings, 14 with one firing, and 7 with three firings. After firing, we used the KHANS technique to reinforce these weak spots using interrupted sutures. Compared to the ROLARR trial, which is, to date, the only randomised controlled trial performed on robotic vs. laparoscopic rectal cancer surgery, our cohort seems to have improved conversion rates (0% vs. 8.1%) and LOS (6.0 vs. 8.0 days) [35].

There are a few limitations to this study, including the selective enrolment of patients for selective diversion. We considered patients with poor functional and nutritional status, immunosuppression, and a poor response to chemoradiotherapy to be at high risk for anastomotic leak, and this technique was not employed in such patients. However, with increasing surgeon experience, even these patients may benefit from this technique. Another potential limitation is that we were unable to do any multivariate or regressive analyses due to the limited number of inclusions. Lastly, the reduction in firings needed for the anastomosis in group B could potentially introduce bias as well, with recent studies showing a correlation between the number of firings and AL, though no robotic cases were included in these studies [36].

## 5. Conclusions

A more selective approach to stoma diversion does not lead to more complications or leaks and can be considered in the treatment of rectal cancer tumours.

## Figures and Tables

**Table 1:** Baseline characteristics. Values are depicted as means ± SD for normally distributed data and as median [Q1–Q3] for non-normally distributed data. A *p*-value of <0.05 is considered significant. Abbreviations: n = number; TME = Total Mesorectal Excision; ASA = American Society of Anaesthesiologists; BMI = Body Mass Index; kg = kilogram; m^2^ = square metre; NA = not applicable; RT = radiotherapy; chemo = chemotherapy; NS = non-significant; ARJ = anorectal junction; T-staging = tumour staging; N-staging = nodal staging; M-staging = metastatic staging; MRI = magnetic resonance imaging; CRM = circumferential resection margin.

		Group A Robot 13–15 (n = 70)	Group B Robot 16–21 (n = 135)	*p*-Value
Sex	Male	45 (64.3%)	86 (63.7%)	1.000
Age at surgery	Median in years [Q1–Q3]	68.00 [62.75–74.25]	69.00 [58.00–76.00]	0.992
ASA	I	10 (14.3%)	22 (16.3%)	0.806
	II	51 (72.9%)	92 (68.1%)	
	III	9 (12.9%)	21 (15.6%)	
BMI	Median in kg/m^2^ [Q1–Q3]	27.00 [24.00–29.00]	26.00 [23.40–29.00]	0.259
	Missing data	0	5	
Type of surgery performed	Anterior resection	70 (100%)	136 (100%)	NA
Previous abdominal surgery	No	47 (68.1%)	94 (75.8%)	0.310
	Total	69	124	
Radiotherapy	None	53 (75.7%)	115 (85.2%)	0.103
	Short-course RT	1 (1.4%)	5 (3.7%)	
	Long-course RT	15 (21.4%)	14 (10.4%)	
	Adjuvant RT	1 (1.4%)	1 (0.7%)	
Chemotherapy	None	31 (44.3%)	77 (57.0%)	NS
	Neoadjuvant	12 (17.1%)	10 (7.4%)	0.011
	Adjuvant	24 (34.3%)	31 (23.0%)	NS
	Both	3 (4.3%)	17 (12.6%)	NS
Tumour height from ARJ on MRI	Median in cm [Q1–Q3]	5.00 [3.00–7.00]	6.00 [4.00–8.00]	0.127
	Missing data	8	12	
Preoperative T-staging	T1	2 (2.9%)	10 (7.4%)	0.009
	T2	40 (58.0%)	47 (34.8%)	
	T3	26 (37.7%)	69 (51.1%)	
	T4	1 (1.4%)	9 (6.7%)	
Preoperative N-staging	N0	46 (65.7%)	67 (50.4%)	0.113
	N1	19 (27.1%)	52 (39.1%)	
	N2	5 (7.1%)	14 (10.5%)	
Preoperative M-staging	M0	68 (97.1%)	127 (94.1%)	0.500
	M1	2 (2.9%)	8 (5.9%)	
MRI-CRM staging	Positive (≤1 mm)	13 (19.7%)	23 (17.2%)	0.397
	Negative (>1 mm)	44 (66.7%)	100 (74.6%)	
	Threatened	9 (13.6%)	11 (8.2%)	
	Missing data	4	1	
Follow-up duration	In months [Q1–Q3]	69.00 [61.75–83.00]	27.00 [19.00–41.00]	<0.001
	Missing data	12	14	

**Table 2:** Operative details and postoperative follow-up. Values are depicted as means ± SD for normally distributed data and as median [Q1–Q3] for non-normally distributed data. A *p*-value of <0.05 is considered significant. Abbreviations: n = number; NA = not applicable; AL = anastomotic leakage; TME = Total Mesorectal Excision; R-staging = Residual tumour staging.

		Group A Robot 13–15 (n = 70)	Group B Robot 16–21 (n = 135)	*p*-Value
Conversion	No	70 (100%)	135 (100%)	NA
Stapler type	None	2 (4.3%)	1 (0.8%)	<0.001
	Laparoscopic	45 (95.7%)	41 (32.0%)	
	Robotic	0 (0%)	86 (67.2%)	
	Missing data	23	7	
Number of linear firings	1	3 (6.7%)	19 (15.2%)	<0.001
	2	15 (33.3%)	89 (71.2%)	
	3	19 (42.2%)	17 (13.6%)	
	4	6 (13.3%)	0 (0%)	
	5	2 (4.4%)	0 (0%)	
	Missing data	25	10	
Operation time	Median in minutes [Q1–Q3]	240.00 [210.00–302.50]	255.00 [240.00–300.00]	0.029
	Missing data	4	16	
Blood loss	Median in mL [Q1–Q3]	0.00 [0.00–20.00]	0.00 [0.00–10.00]	0.635
	Missing data	15	24	
Length of stay	Median in days [Q1–Q3]	6.00 [5.00–9.25]	6.00 [4.00–9.00]	0.292
Readmission < 31 days	No	58 (84.1%)	112 (83.0%)	1.000
	Missing data	1	0	
Reoperation < 31 days	No	66 (97.1%)	126 (93.3%)	0.342
	Missing data	2	0	
Complication grade (Clavien Dindo)	No complication	39 (55.7%)	52 (38.5%)	0.117
	Grade 1	0 (0%)	6 (4.4%)	
	Grade 2	27 (38.6%)	56 (41.5%)	
	Grade 3a	2 (2.9%)	9 (6.7%)	
	Grade 3b	2 (2.9%)	8 (5.9%)	
	Grade 4	0 (0%)	3 (2.2%)	
	Grade 5	0 (0%)	1 (0.7%)	
Pathological Tumour stage	T0	1 (1.5%)	3 (2.3%)	0.623
	T1	8 (11.9%)	8 (6.1%)	
	T2	26 (38.8%)	55 (42.0%)	
	T3	29 (43.3%)	58 (44.3%)	
	T4a	0 (0%)	3 (2.3%)	
	T4b	3 (4.5%)	4 (3.1%)	
	Missing data	3	4	
Pathological R-staging	R0	67 (95.7%)	128 (94.8%)	1.000
	R1	3 (4.3%)	7 (5.2%)	
<91-day mortality	No	69 (98.6%)	134 (99.3%)	1.000

**Table 3:** Anastomotic leakages. Values are depicted as means ± SD for normally distributed data and as median [Q1–Q3] for non-normally distributed data. A *p*-value of <0.05 is considered significant. Abbreviations: n = number; Lap = laparoscopic; TME = Total Mesorectal Excision; AL = anastomotic leakage; ISREC = International Study Group of Rectal Cancer.

		Group A Robot 13–15 (n = 70)	Group B Robot 16–21 (n = 135)	*p*-Value
Complications of anastomotic leakage	No	60 (88.2%)	111 (82.2%)	0.312
	Yes	8 (11.8%)	24 (17.8%)	
		Early: 3 (4.4%)	Early: 18 (13.3%)	
		Late: 5 (7.4%)	Late: 6 (4.4%)	
	Missing data	2	0	
Time until AL	Median in days [Q1–Q3]	31.50 [3.75–96.25]	8.50 [4.00–65.50]	0.542
	Total	8	24	
Specification: early leaks	Diverted	3/3	15/18	1.000
	Not diverted	0/0	3/18	
ISREC classification leakage	A	4/8	5/24	0.326
	B	3/8	12/24	
	C	1/8	7/24	
ISREC class C specifics	Reoperation	1	6	1.000
	Radiological drainage	0	1	
	Death	0	0	
Specification: ISREC class B	Diverted	3/3	11/12	1.000
	Not diverted	0/3	1/12	
Specification: ISREC class C	Diverted	1/1	5/7	1.000
	Not diverted	0/1	2/7	

**Table 4:** Stoma variables. Values are depicted as means ± SD for normally distributed data and as median [Q1–Q3] for non-normally distributed data. A *p*-value of <0.05 is considered significant. Abbreviations: n = number; Lap = laparoscopic; TME = Total Mesorectal Excision; LOS = length of stay.

		Group A Robot 13–15 (n = 70)	Group B Robot 16–21 (n = 135)	*p*-Value
Stoma type	None	7 (10.0%)	37 (27.4%)	0.004
	Ileostomy temporary	63 (90.0%)	98 (72.6%)	
Stoma closed	No	11 (17.5%)	21 (21.4%)	0.686
Complications of stoma closure	No	27 (60.0%)	31 (49.2%)	0.329
	Missing data	7	14	
LOS stoma closure	Median in days [Q1–Q3]	4.00 [3.00–7.00]	4.00 [4.00–6.25]	0.373
	Missing data	21	73	
Readmission after stoma closure	No	50 (96.2%)	70 (90.9%)	0.312
Reoperation after stoma closure	No	50 (96.2%)	75 (97.4%)	1.000
Time until closure	Median in days [Q1–Q3]	284.00 [183.25–378.75]	234.00 [148.00–332.00]	0.307
	Missing data	18	68	
Reasons for delayed/no reversal	Adjuvant chemo	10 (58.8%)	11 (40.7%)	0.054
	Adjuvant therapy	0 (0%)	2 (7.4%)	
	Anastomotic leak	3 (17.6%)	6 (22.2%)	
	Other treatment	4 (23.5%)	1 (3.7%)	
	Complications	0 (0%)	1 (3.7%)	
	COVID-19	0 (0%)	6 (22.3%)	
	Total	17	27	
Secondary stoma	No	65 (97.0%)	126 (94.7%)	0.721
	Missing data	3	2	
Stoma-free at 1 year	No	19 (28.4%)	36 (28.1%)	1.000
	Missing data	3	7	
Stoma-free at end of follow-up	No	10 (14.9%)	32 (24.1%)	0.146
	Missing data	3	2	

**Table 5:** Within-group subanalysis of group B, comparing diverting stoma patients with non-diverting stoma patients. Values are depicted as means ± SD for normally distributed data and as median [Q1–Q3] for non-normally distributed data. A *p*-value of <0.05 is considered significant. Abbreviations: n = number; ASA = American Society of Anaesthesiologists; BMI = Body Mass Index; kg = kilogram; m^2^ = square metre; NA = not applicable; RT = radiotherapy; chemo = chemotherapy; NS = non-significant; ARJ = anorectal junction; T-staging = tumour staging; N-staging = nodal staging; M-staging = metastatic staging; MRI = magnetic resonance imaging; CRM = circumferential resection margin; AL = anastomotic leakage; ISREC = International Study Group of Rectal Cancer.

		No-Stoma Group B (n = 37)	Diverting Stoma Group B (n = 98)	*p*-Value
Sex	Male	14 (37.8%)	72 (73.5%)	<0.001
Age at surgery	Median in years [Q1–Q3]	65.00 [51.00–76.50]	70.00 [61.00–76.25]	0.151
ASA	I	9 (24.3%)	13 (13.3%)	0.253
	II	24 (64.9%)	68 (69.4%)	
	III	4 (10.8%)	17 (17.3%)	
BMI	Median in kg/m^2^ [Q1–Q3]	27.00 [23.40–29.00]	25.90 [23.40–29.00]	0.409
	Missing data	2	3	
Previous abdominal surgery	No	22 (71.0%)	72 (77.4%)	0.629
	Missing data	6	5	
Radiotherapy	None	35 (94.6%)	80 (81.6%)	0.046
	Short-course RT	0 (0%)	5 (5.1%)	
	Long-course RT	1 (2.7%)	13 (13.3%)	
	Adjuvant RT	1 (2.7%)	0 (0%)	
Chemotherapy	None	24 (64.9%)	53 (54.1%)	0.011
	Neoadjuvant	0 (0%)	10 (10.2%)	
	Adjuvant	12 (32.4%)	19 (19.4%)	
	Both	1 (2.7%)	16 (16.3%)	
Tumour height from ARJ on MRI	Median in cm [Q1–Q3]	8.00 [5.63–10.0]	5.00 [3.00–7.00]	<0.001
	Missing data	5	7	
Preoperative T-staging	T1	7 (18.9%)	3 (3.1%)	0.021
	T2	12 (32.4%)	35 (35.7%)	
	T3	17 (45.9%)	52 (53.1%)	
	T4	1 (2.7%)	8 (8.2%)	
Preoperative N-staging	N0	19 (52.8%)	48 (49.5%)	0.564
	N1	15 (41.7%)	37 (38.1%)	
	N2	2 (5.6%)	12 (12.4%)	
Preoperative M-staging	M0	37 (100%)	90 (91.8%)	0.106
	M1	0 (0%)	8 (8.2%)	
MRI-CRM staging	Positive (≤1 mm)	2 (5.6%)	21 (21.4%)	0.022
	Negative (>1 mm)	33 (91.7%)	67 (68.4%)	
	Threatened	1 (2.8%)	10 (10.2%)	
	Missing data	1	0	
Operation time	Median in minutes [Q1–Q3]	240 [215–290]	260 [240–305]	0.004
	Missing data	8	8	
Length of stay	Median in days [Q1–Q3]	4.00 [4.00–6.00]	6.00 [5.00–10.25]	<0.001
Readmission < 31 days	No	33 (89.2%)	79 (80.6%)	0.309
Reoperation < 31 days	No	35 (94.6%)	91 (92.9%)	1.000
Complication grade (Clavien Dindo)	No complication	20 (54.1%)	32 (32.7%)	0.154
	Grade 1	3 (8.1%)	3 (3.1%)	
	Grade 2	10 (27.0%)	46 (46.9%)	
	Grade 3a	2 (5.4%)	7 (7.1%)	
	Grade 3b	2 (5.4%)	6 (6.1%)	
	Grade 4	0 (0%)	3 (3.1%)	
	Grade 5	0 (0%)	1 (1.0%)	
Pathological Tumour stage	T0	0 (0%)	3 (3.2%)	0.164
	T1	5 (13.9%)	3 (3.2%)	
	T2	12 (33.3%)	43 (45.3%)	
	T3	18 (50.0%)	40 (42.1%)	
	T4a	0 (0%)	3 (3.2%)	
	T4b	1 (2.8%)	3 (3.2%)	
	Missing data	1	3	
Complications of anastomotic leakage	No	34 (91.9%)	77 (78.6%)	0.081
	Yes	3 (8.1%)	21 (21.4%)	
		Early: 3 (100%)	Early: 15 (71.4%)	
		Late: 0 (0%)	Late: 6 (28.6%)	
ISREC classification leakage	A	0/3	5/21	0.401
	B	1/3	11/21	
	C	2/3	5/21	
ISREC class C specifics	Reoperation	2	4	1.000
	Radiological drainage	0	1	
	Death	0	0	
Pathological R-staging	R0	35 (94.6%)	93 (94.9%)	1.000
	R1	2 (5.4%)	5 (5.1%)	
<91-day mortality	No	37 (100%)	97 (99.0%)	1.000

## Data Availability

Upon reasonable request, anonymised data can be made available as appropriate.
